# Checks and Balances in Bacterial Cell Division

**DOI:** 10.1128/mBio.00149-19

**Published:** 2019-02-26

**Authors:** Tanneke den Blaauwen, Joen Luirink

**Affiliations:** a Bacterial Cell Biology & Physiology, Swammerdam Institute for Life Sciences, University of Amsterdam, Amsterdam, The Netherlands; b Department of Molecular Microbiology, Institute for Molecules Medicines and Systems (AIMMS), Vrije Universiteit, Amsterdam, The Netherlands

**Keywords:** *Escherichia coli*, cell division, divisome, peptidoglycan

## Abstract

Assembly of the division machinery in Gram-negative and Gram-positive bacteria occurs in two time-dependent steps. First, the FtsZ proto-ring localizes at midcell including some FtsN molecules. Subsequently, the proteins that catalyze and regulate septal peptidoglycan (PG) synthesis are recruited including among others, the FtsBLQ-PB1B-FtsW-PBP3 complex. Further accumulation of FtsN finally allows initiation of cell division. It was known that FtsA and FtsQLB somehow prevented this initiation. Recently, A. Boes, S. Olatunji, E. Breukink, and M. Terrak (mBio 10:e01912-18, 2019, https://doi.org/10.1128/mBio.01912-18) reported that this is caused by inhibition of the activity of the PG synthases by FtsBLQ, which has to be outcompeted by accumulation of the PBP1b activating FtsN. This supports a central structural as well as regulatory role for the FtsBLQ protein complex that is conserved only in prokaryotes, making it an attractive target for antibiotic development.

## Abstract (precis)

Assembly of the division machinery in Gram-negative and Gram-positive bacteria occurs in two time-dependent steps. First, the FtsZ proto-ring localizes at midcell including some FtsN molecules.

## COMMENTARY

Cell division in Gram-negative bacteria is orchestrated in time and place by many periplasmic proteins and by at least 12 essential membrane (membrane-associated) proteins that are recruited to the bacterial midcell plane. Together they form an extended assembly referred to as the divisome that ensures proper cell constriction, septal peptidoglycan (PG) synthesis, and ultimately cell separation (1). Assembly starts with formation of the Z-ring and ends with the arrival of FtsN that prompts septal PG synthesis. The enzymatic activities of the individual subunits, as well as their interactions and assembly into a functional divisome, are prime targets for intervention through antimicrobials.

At the heart of the bacterial divisome, the bitopic membrane proteins FtsQ, FtsB, and FtsL form a subcomplex (2), the function of which has remained unclear for years. In the hierarchical divisome assembly process, the FtsBLQ complex is recruited by FtsK and in turn recruits FtsW-PBP3, but two-hybrid analyses have suggested that FtsBLQ interacts with many other cell division proteins as well (3, 4). On the basis of these observations and the absence of evidence for a clear catalytic function, FtsBLQ has been considered a molecular connector that coordinates a multitude of transient interactions in the divisome (5) although more-recent evidence has suggested a regulatory role for FtsBLQ in the initiation of septal PG synthesis (6–8). New data from Boes and colleagues (9) demonstrate that FtsBLQ directly inhibits key enzymes in septal PG synthesis until the time is right to divide.

The connections of FtsBLQ have been heavily investigated to define various interactions in molecular detail and to pinpoint potential antimicrobial targets. The periplasmic domain of FtsQ consists of two subdomains, termed α and β (10). Together with the transmembrane domain (TMD), the membrane-proximal α domain is required for recruitment by FtsK, although other interactions have been ascribed to this domain as well. This α domain has homology to a so-called polypeptide transport-associated (POTRA) domain, a conserved chaperone moiety in various transporter proteins (11). The β domain has also been implicated in multiple interactions, including those with the C-terminal regions of FtsB and FtsL (12–16). FtsB and FtsL are small, have similar domain architectures, and can form a subcomplex independent of FtsQ (17), although recruitment to the divisome does require FtsQ (2). The TMD and membrane-proximal coiled-coil regions of FtsB and FtsL are connected in a tetramer conformation, as a recent structural study suggested (18).

The next recruit to the divisome is FtsW, a complex multipass inner membrane protein that is the bottle neck for lipid II feed through the division machinery, as illustrated by the inability of divisome member and flippase MurJ to flip lipid II across the inner membrane in the absence of active FtsW (19). FtsW was recently suggested to also have PG transglycosylase activity, the activation of which appeared dependent on the cell division PG transpeptidase PBP3 that is always found associated with FtsW (20). PBP3 in turn, has affinity for another PG synthase, PBP1b, as shown by *in vivo* cross-linking and SPR (21).

While the late recruit FtsN is not a PG synthase itself, it does interact with the PG synthases PBP3, PBP1b, and MgtA (3, 22, 23). Like FtsQ, FtsB, and FtsL, FtsN is a bitopic membrane protein. It has a short cytoplasmic domain that can interact with FtsA (24), a TMD, and a long periplasmic domain that ends in a PG binding domain (25). The interaction with FtsA in the proto-ring, the PG synthases, and the PG layer itself provide FtsN with a shortcut to signal the readiness of the division machinery and to promote initiation of septal PG synthesis.

Importantly, initiation of septal PG synthesis was recently found to be linked not only to FtsA but also to the presence of FtsBLQ (6–8), indicating that this subcomplex is more than a mere scaffold for divisome assembly. Mutations in FtsL or FtsB allowed the (partial) bypass of cell division proteins ZipA, FtsA, FtsK, and FtsN. The mutations clustered at the end of the coiled-coil regions (around residues 56 in FtsB and 93 in FtsL) that was referred to as the constriction control domain (CCD). These data together suggested that both FtsA and FtsBLQ need to assume an on state for the initiation of septal PG synthesis and consequent cell constriction. However, it was not clear what exactly this on state represented.

The study of Boes and coworkers (9) published recently in *mBio* now provides mechanistic insight in the inhibitory and stimulatory roles of FtsBLQ and FtsN, respectively, in the regulation and timing of septal PG synthesis. Various late divisome proteins were expressed, purified, and combined to study PG transpeptidase and transglycosylase activity *in vitro*. Some of the suggested interactions of FtsQBL and FtsN with other divisome proteins were confirmed biochemically. This is commendable in itself, given the challenge of coexpressing and purifying essential integral membrane proteins. A six-protein complex could be purified consisting of FtsBLQ, FtsW, PBP1b, and PBP3. The scientists also discovered that FtsN interacts with PBP1b to stimulate its GTase activity rather than to interact with FtsBLQ directly.

Most strikingly, FtsBLQ was shown to inhibit the GTase activity of PBP1b, and a direct connection between PBP1b and FtsBLQ was demonstrated. Moreover, using an artificial substrate, FtsBLQ was shown to also inhibit the TPase activity of PBP3, which consequently inhibits the activity of FtsW. These experiments support the hypothesis that FtsBLQ keeps septal PG synthesis in check until the cell is ready for it but reveal that the FtsBLQ role is more direct than anticipated.

In finer detail, the activity of PBP3 was primarily inhibited by FtsQ, whereas FtsBL appeared to interact with PBP1b and inhibit its activity. This is consistent with the fact that suppressor mutations that bypass the PBP1b stimulator FtsN were found in FtsBL but not in FtsQ. Analysis of purified FtsQBL complexes with the suppressor mutations in the CCD of either FtsB or FtsL further suggested that FtsL is in fact the specific inhibitor of PBP1b.

**FIG 1:**
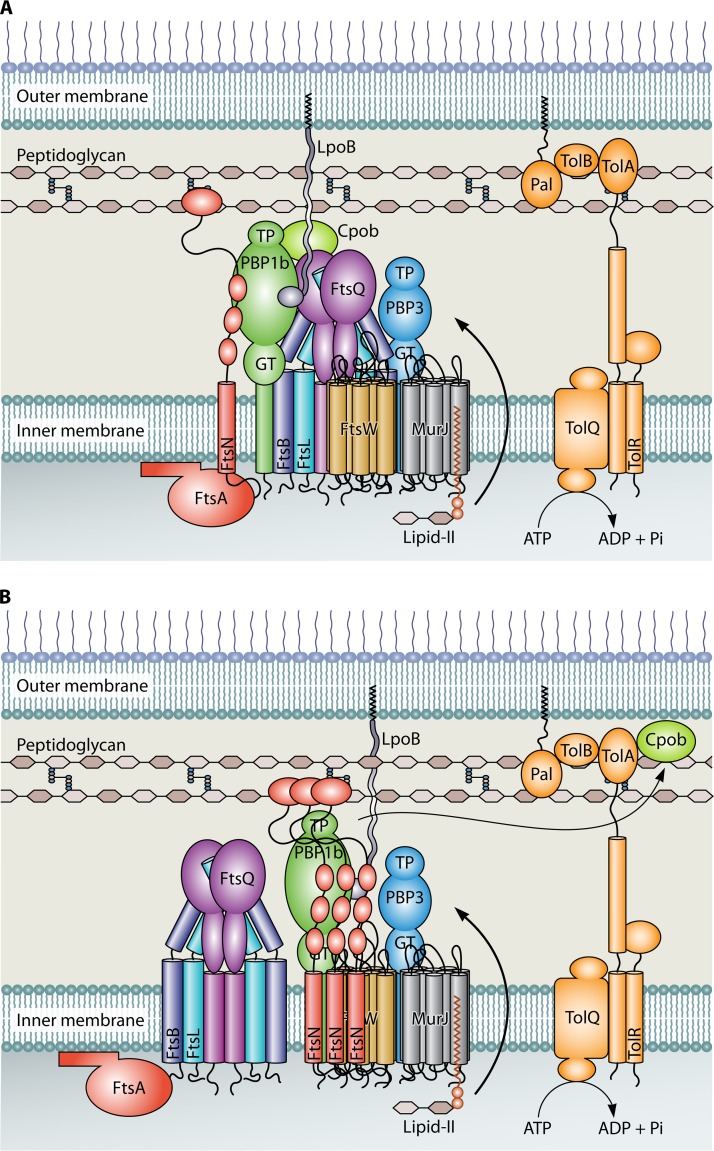
Schematic representation of the possible interactions and activation in the divisome. Panel A shows the divisome when FtsN is initially recruited by FtsA (31). For the model, only the relevant divisome proteins are shown. This switches FtsA to the on state, and further accumulation of FtsN allows successful competition with FtsBLQ for the peptidoglycan synthetic complex shown in panel B. FtsN activates PBP1B, which becomes hyperactive due to the absence of CpoB. The concomitant abolishment of PBP3 inhibition allows FtsW activity and the initiation of septal PG synthesis. Since none of the PG hydrolases are included in the model, at this time, we assume that the regulation of septation will be more complex than shown here.

Thus, the initiation of septal PG synthesis is in part regulated through antagonistic effects of FtsBLQ and FtsN on the activity of PBP1b, the balance of which will be tipped by recruitment of FtsN at the septum where the core PG synthase FtsW-PBP3-PBP1b is gathered (Fig. 1). Adding to the complexity of this regulation, FtsW and CpoB, via LpoB, also suppress the activity of PBP1B (26, 27). However, at midcell, CpoB is likely removed from PBP1B by TolA, allowing PBP1b to become much more active (26, 28).

In any case, increasing numbers of FtsN at midcell appear sufficient to outcompete FtsBLQ, perhaps also driving it away from PBP3, which will alleviate inhibition of the PBP3 activity and therefore also allows FtsW to become active. Inhibition of the septal synthesis complex is then abrogated, and the PBP1B activity is enhanced, allowing fast local PG synthesis. Because the midcell position is now established and PG synthesis proceeds unrestricted, the proto-ring and synthetic machinery do not have to remain associated, as recently illustrated by superresolution microscopy (29).

Thus, FtsBLQ is initially needed for the assembly of the divisome and secondly to halt its activity until it is safe to allow septal PG synthesis. Interference with FtsBLQ will either prevent assembly of the divisome or result in premature division and potentially lysis. Molecules that compromise the assembly of the FtsBLQ complex will likely accomplish both. The complex has conserved elements in the ESKAPE organisms and will probably have a similar function in these pathogenic strains. Although inhibition of protein interactions is usually not the first choice of antimicrobial strategy because of relatively easy resistance development, one could also argue that given the multitude of interactions of FtsBLQ, multiple protein partners will have to adapt for resistance to develop. A more technical challenge in the development of small-molecule modulators of protein interactions lies in the often large, shallow interaction interfaces that connect proteins (30). However, this may be different for the “interaction-prone” divisome proteins. Interestingly, a recent crystal structure of FtsB bound to the β domain of FtsQ shows that the contact is limited to the conserved residues 64 to 87 of FtsB that interact with an equally conserved region around residue 248 in FtsQ (13). Other features that qualify FtsBLQ as an interesting target for this approach include their low abundance, relatively accessible location, and the absence of human homologues.

Structure-based design of modulating molecules will be required to underpin this strategy. Although partial crystal structures of most individual key players are available, more insight in the size and structure of interaction interfaces needs to be generated. The study by Boes and coworkers (9) suggests that larger late divisome complexes can be purified in sufficient amounts for single particle analysis by transmission electron microscopy. Complemented with available crystal structures, this should allow a rational design of modulators that interfere with protein interactions in the divisome.
