# Erratum for den Blaauwen and Luirink, “Checks and Balances in Bacterial Cell Division”

**DOI:** 10.1128/mBio.00615-19

**Published:** 2019-04-16

**Authors:** Tanneke den Blaauwen, Joen Luirink

**Affiliations:** a Bacterial Cell Biology & Physiology, Swammerdam Institute for Life Sciences, University of Amsterdam, Amsterdam, The Netherlands; b Department of Molecular Microbiology, Institute for Molecules Medicines and Systems (AIMMS), Vrije Universiteit, Amsterdam, The Netherlands

## ERRATUM

Volume 10, issue 1, e00149-19, 2019, https://doi.org/10.1128/mBio.00149-19. After review of Fig. 1, we noticed that the pedestal domain of PBP3 was erroneously annotated as GT. The GT label was intended to be placed on FtsW.

